# Supplementary figures and images for: Radiation Hormesis in Barley Manifests as Changes in Growth Dynamics Coordinated with the Expression of PM19L-like, CML31-like, and AOS2-like
Source: Int J Mol Sci. 2024 Jan 12;25(2):974. doi: 10.3390/ijms25020974 (PMC10815718; doi:10.3390/ijms25020974)

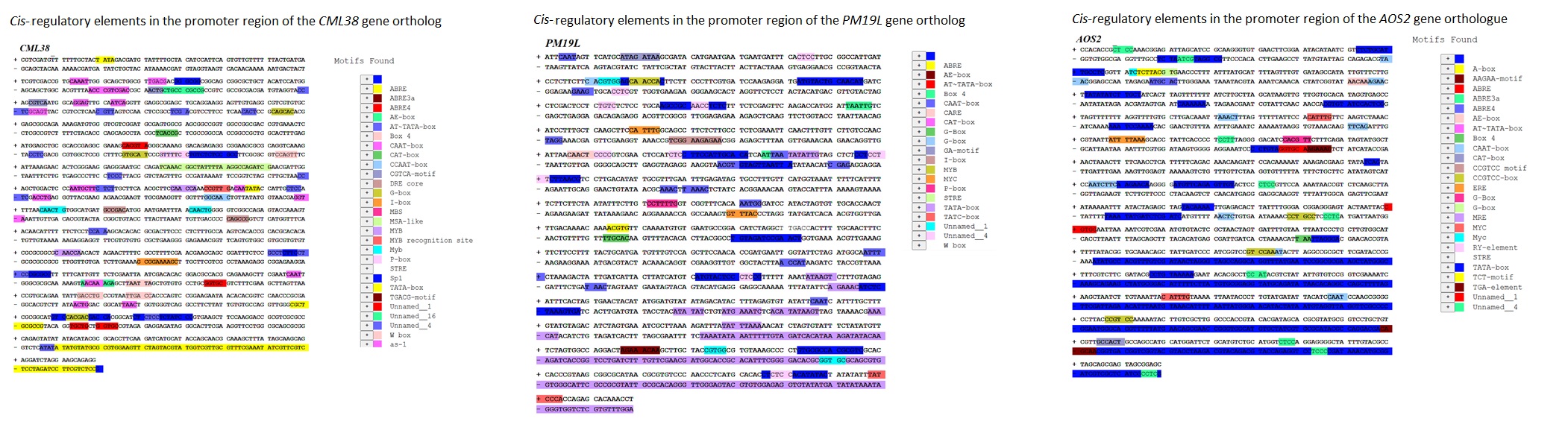

Supplement: Supplementary file 1 [file ijms-25-00974-s001.zip › SF1 Regulatory elements in the promoter regions of candidate genes.jpg]
